# Comparative evaluation of bags used to collect samples of oxygen, carbon dioxide, and methane for use in open-circuit indirect calorimetry

**DOI:** 10.3168/jdsc.2025-0774

**Published:** 2025-06-18

**Authors:** A.K. Neff, K.K. Buse, A.L. Carroll, T.M. Brown-Brandl, P.J. Kononoff

**Affiliations:** 1 Department of Animal Science, University of Nebraska–Lincoln, Lincoln, NE 68503; 2 Department of Biological Systems Engineering, University of Nebraska–Lincoln, Lincoln 68583

## Abstract

Methane (CH_4_), carbon dioxide (CO_2_), and oxygen (O_2_) are the major gases produced by dairy cattle as a result of rumen fermentation and metabolism, and thus, their concentrations are frequently measured as a way of estimating heat production and energy metabolism. A well-utilized method of measuring gas consumption and production to estimate heat production is indirect calorimetry, which requires bags to retain the sampled gases until analysis. The objective of this study was to determine the ability of a polyvinyl fluoride gas bag (PF) and a multilayer fabrication gas bag containing an aluminum layer (NAP) to maintain respiratory gas composition in comparison to a polyethylene terephthalate bag (PET). For experiments 1 and 2, respiratory gases were collected from 6 multiparous, lactating Jersey cows using headbox-style indirect calorimeters. During experiment 1, PF and PET were used to sample respired gases of the headboxes for each cow. Experiment 2 used a similar design to compare NAP and PET. In both experiments, respired air was sampled into the gas bags for 5 h before being analyzed immediately to determine the concentrations of the gases. All bags were reanalyzed for gas concentrations at 24 and 72 h after sampling to determine the ability to maintain the concentration of gas over a 3-d period. In experiment 1, a tendency was observed for time by bag material interaction, as PET retained a consistent concentration of O_2_ (20.37% ± 0.020%) over the 72-h period, whereas the O_2_ concentration in PF increased from 20.37% to 20.47% ± 0.02%. Similarly for CO_2_, PET maintained a consistent concentration of 0.653% ± 0.020% from hour 0 to 72, whereas PF significantly decreased from 0.65% to 0.55% ± 0.02%. No interactions between bag material and time occurred for CH_4_ across 72 h for PET and PF, averaging 0.0421% ± 0.005%, respectively. In experiment 2, comparing NAP and PET, no bag material, time, or bag material by time interactions were observed for O_2_ concentration, CO_2_ concentration, or CH_4_ concentration, averaging 20.37% ± 0.020% O_2_, 0.648% ± 0.025% CO_2_, and 0.0377% ± 0.0100% CH_4_ between treatments. Results indicate a significant difference in gas concentrations over time for PF relative to PET, and no difference was observed in gas concentrations for NAP in comparison to the PET bag. Thus, gas bag material type and time to gas analysis should be considered to effectively characterize respiratory gas composition.

## Highlights

• Gas sample storage method affects measures of O_2_, CO_2_, and CH_4_.
• Concentration of CO_2_ was decreased in the PF bag within 24 hours of storage.
• Gas composition maintained for 72 hours in the PET bag.
• Gas composition maintained for 72 hours in multilayer bag with aluminum.

## Graphical Abstract

**Figure:**
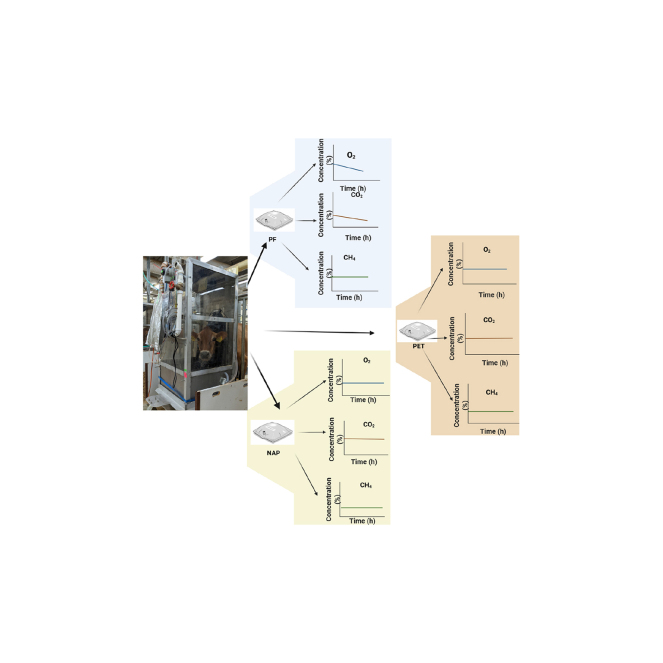
**Summary:** During feed metabolism, ruminants consume O_2_ and produce CO_2_ and CH_4_; thus, accurate analyses of these gases after capture are not only integral to quantifying environmental impact, but also describing nutrient utilization. This article recounts 2 experiments evaluating the ability of gas bags made of polyvinyl fluoride (PF) and multilayer fabrication with aluminum (NAP) to retain gas concentrations over a 72-hour period compared with a polyethylene terephthalate (PET) control. The results from the experiment determined that the PF bags initially contained similar gas concentrations relative to the PET, but the PF bag did not retain similar concentrations of O_2_ and CO_2_ over the 72-hour period, leading to significant treatment by time interactions for experiment 1. In experiment 2, the NAP bag and the PET control bag initially contained and maintained similar concentrations of O_2_, CO_2_, and CH_4_. This resulted in no significant interactions in regard to the NAP in comparison to the PET over time. These results suggest PF does not maintain respiratory gas composition over time and that the NAP bag is a comparable gas bag to the PET gas bag.

Indirect calorimetry is a technique used to study energy utilization by estimating body heat production (**HP**), a loss of energy that accounts for ~28% of total energy intake (Carroll et al., 2024). To indirectly estimate HP, investigators must measure O_2_ consumed as well as CO_2_ and CH_4_ produced by the animal (Reynolds et al., 2019). These data, along with urinary N content, are usually integrated into an equation outlined by Brouwer (1965) to estimate HP.

To accurately measure the volume of gases consumed and produced, samples of ambient and respired air must be collected and analyzed (Reynolds et al., 2019). Materials and fabrication methods used to construct sampling bags must ensure that the composition of collected gases remains unchanged until analysis. This presents a challenge because different gases have unique physical and chemical properties that may interact with bag materials. For example, CO_2_ is chemically stable but has been shown to diffuse through certain polymer-based sampling bags, such as those made from Tedlar (DuPont, Wilmington, DE), potentially compromising concentration measurements over time (Sherman et al., 2004). Although oxygen (O_2_) is chemically stable in the atmosphere, it may contribute to the degradation of certain odorous compounds in gas samples through slow oxidation or interaction with reactive compounds or bag materials, potentially leading to odor concentration loss during storage (van Harreveld, 2003). These behaviors make the collection and analysis of gases like CO_2_ and O_2_ challenging, as both are subject to physical diffusion and possible chemical transformation. Constructing sample bags from low-permeability or chemically inert materials may help to mitigate these effects. Methane (CH_4_), by contrast, is chemically stable and generally less reactive than gases such as O_2_, making it less prone to degradation during storage. However, like other small molecules, CH_4_ may still diffuse through some polymer sampling bags over time, depending on the material and storage conditions **(**Arrhenius et al., 2016). Any interaction between the gases and the sampling bag materials may lead to measurement errors in key variables such as the estimation of HP. Therefore, the objective of this study was to evaluate the stability of gas composition over time in sampling bags made from different materials and compare their performance.

Using 2 completely randomized designs, separate experiments were conducted to evaluate gases collected and stored over time using 3 types of gas storage bags that differed in material and method of fabrication. The first bag, which is no longer commercially available but has been used in previous studies involving indirect calorimetry (Foth et al., 2015), was constructed out of polyethylene terephthalate (**PET**; 40 L, LAM-JAPCON-NSE, Pollution Measurement Corp., Oak Park, IL). The second was constructed out of polyvinyl fluoride (**PF**; Tedlar, DuPont, Wilmington, DE; 40 L, 61 × 61 cm, RKI Instruments, 81-1155, Union City, CA). The third bag used in our study was a multilayer fabrication (**NAP**) containing 0.015 mm nylon (outer layer), polyethylene, 0.0076 mm aluminum foil, and aluminum coated 0.051 mm polyethylene (inner layer; 40 L, 61 × 62 cm, RESTEK, Bellefonte, PA). In both experiments, PET served as a control and was compared with PF in experiment 1 and NAP in experiment 2.

All animal care and experimental procedures were approved by the University of Nebraska–Lincoln Animal Care and Use Committee. Six multiparous lactating Jersey cows (422 ± 45.9 kg BW, 129.3 ± 15 DIM, 29.2 ± 2.44 kg/d milk yield, 19.1 ± 2.2 kg/d DMI) were used in both experiment 1 and 2. Ambient air in the research facility averaged 20.89% O_2_, 0.097% CO_2_, and 0.0047% CH_4_. The cows were housed in tiestalls with rubber mats in a temperature-controlled (20°C) barn with continuous access to water at the Dairy Metabolism facility located in the Department of Animal Science on the University of Nebraska–Lincoln East Campus (Lincoln, NE). The cows were milked twice a day at 0700 and 1800 h and fed a TMR once daily at 0930 h.

For each experiment, one control bag (PET) and one treatment bag (either PF or NAP) was attached to each of the 6 headbox-type indirect calorimeters as described previously (Foth et al., 2015; McLain et al., 2021; Carroll et al., 2023). From each headbox, continuous samples of respiratory and eructed enteric gases were collected into the treatment gas bags (PET and PF or PET and NAP) through 2 split-line glass tube rotameters set to 55 mL/min to ensure proper gas collection (Model 1350E Sho-Rate “50,” Brooks Instruments, Hatfield, PA). Cows were placed into the head chambers at feeding, and gases were sampled for 5 h. Line pressure was measured using a U-tube manometer (item #1221-8, Park Supply of America Inc., Minneapolis, MN). Gas flow through the headbox was measured using mass flow meters set to ~0.68 standard cubic meters per minute (MCW Whisper, Alicat Scientific, Tucson, AZ) and corrected to standard temperature and pressure (0°C, 760 mm Hg).

The gas contained in all bags was analyzed according to the method of Nienaber and Maddy (1985) for O_2_, CO_2_, and CH_4_ using an Emerson X-stream 3-channel analyzer (Solon, OH) with paramagnetic O_2_ analyzer with a suppressed zero range of 20% to 21% O_2_ and a dual-channel infrared analyzer for CO_2_ and CH_4_. A portion of gas from the bags was immediately analyzed upon removal from the headboxes (0 h), and the same bags were subsequently reanalyzed for these 3 gases at 24 and 72 h. This procedure was duplicated for each experiment. Data were analyzed by experiment using the GLIMMIX procedure in SAS (9.4; https://www.sas.com/en_us/home.html). The model contained fixed effects of bag material, time, and the interaction of bag material with time. Random effects included cow and repeated effect of time with an autoregressive covariance structure. All data are presented as LSM ± largest SE. Significance was declared with a *P*-value ≤0.05 and tendencies at *P*-value >0.05 and ≤0.10.

The present experiments were designed to test the stability of gas concentrations when stored in 2 different types of gas sampling bags and compare them to a bag previously used but no longer available. In experiment 1, gas contained in PET was compared with PF over time, and an interaction between bag material and time tended to be observed for O_2_ (*P* = 0.06). At 0 h, O_2_ concentration was similar between PET and PF, but over time, concentration of O_2_ in PF increased by 0.03 and 0.10% after 24 and 72 h, respectively (Figure 1). An interaction (*P* < 0.01) between bag material (PET and PF) and time was also observed for CO_2_. In contrast to what was observed with O_2_, PF contained 0.02% and 0.10% less CO_2_ at 24 and 72 h, respectively. The percentage of CH_4_ was lower (*P* = 0.04) in PF relative to PET but did not differ over time (*P* = 0.94), and no interaction effect was observed (*P* = 0.25) between these 2 factors. Thus, results indicate that the differences in materials used to construct these bags was responsible for the observed differences in O_2_ and CO_2_. Furthermore, the increase over time in the ratio of O_2_ to CO_2_ in PF relative to PET suggests that the PF bag could not prevent ambient gases from permeating the surface, increasing the relative O_2_ concentrations with CO_2_ diffusing from the bag (Ajhar et al., 2010). Previous observations indicated the CO_2_ diffusion coefficient across a 3-L 12.7 × 12.7-cm PF bag was 0.0000038% CO_2_ per hour (Schulz et al., 2004) equating to 0.000274% difference over 72 h. We observed a difference of ~0.1024% at 72 h between PET and PF, and thus, the rate of decline in the 40-L PF bag was 374 times larger than that of the 3-L PF bag. According to Fick's law, the increased rate of CO_2_ loss may be attributed to the greater surface area of the 40 L bag relative to the 3-L bag, which enhances diffusion potential over time. These findings not only indicate that PF bags diffuse ambient air, but that the size of the bag itself may increase the rate of decline over time.

**Figure 1:**
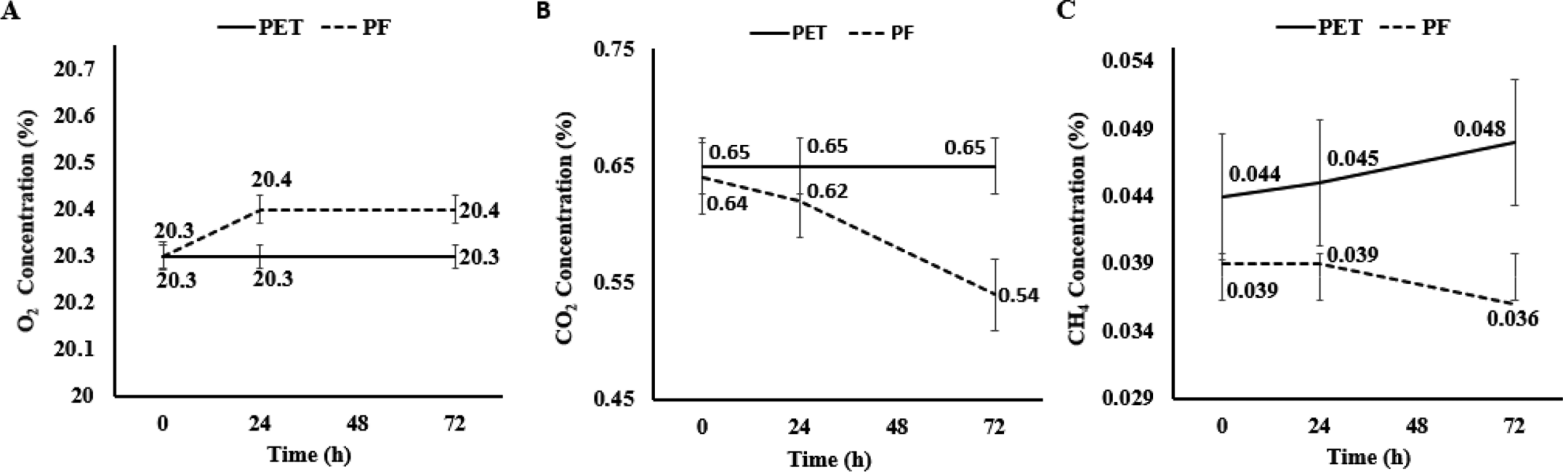
The percentage of O_2_ (A), CO_2_ (B), and CH_4_ (C) contained in a polyethylene terephthalate (PET) and polyvinyl fluoride (PF) bags plotted over time. For O_2_, a significant difference was observed for bag material (*P* = 0.03), time (*P* = 0.02), and a tendency for an interaction between bag material and time (*P* = 0.06). A significant difference in bag material, time, and the interaction between bag material and time was observed for CO_2_ (*P* ≤ 0.01). Methane differed (*P* < 0.01) by bag material, but we observed no significant difference with time (*P* = 0.94) or the interaction of bag material and time (*P* = 0.25). Error bars indicate SEM.

These observations are important because differences in gas composition (O_2_, CO_2_, and CH_4_ percentage) will affect HP estimated by the Brouwer (1965) equation. To illustrate this, using the observations collected at 72 h, we estimated total HP. The gas concentrations were then coupled with assumptions regarding the calculation of total gas production (L/d), including a flow rate of 0.68 standard cubic meters per minute, dew-point temperature of 20°C, a barometric pressure of 101,592 Pa Hg, and an ambient air gas concentration of 20.95% O_2_, 0.042% CO_2_, and 0.00019% CH_4_ (NOAA, 2024). The gas composition in PF at 72 h underestimated HP by 4.4 Mcal/d relative to PET. Assuming 1 kg of milk requires 0.70 Mcal of NEL, the magnitude of the differences in HP are energetically equivalent to underestimating milk yield by 6.3 kg/d (Hutchison, 2019). As a result, the gas bag material type reduces estimates of total gas production, leading to differences in the ability to characterize the HP of ruminants, which leads us to the conclusion that the PF bag was unsuitable for composite sample collection for indirect calorimetry.

In experiment 2, we compared PET to NAP, and the percentage of individual gases in the bag was not affected by bag material (*P* ≥ 0.36) or time (*P* ≥ 0.69). On average, the percentages of O_2_, CO_2_, and CH_4_ contained in the bag were observed to be 20.37% ± 0.020%, 0.648% ± 0.025%, and 0.0375% ± 0.0100%, respectively (Figure 2). No interactions were observed between bag material and time for any gas (*P* ≥ 0.61). Overall, our results serve as supporting evidence that the NAP bags can be used in place of traditional PET bags. However, future research should evaluate time intervals below 24 h and extended time after sampling to better characterize changes in gas concentration. Due to the rapid expansion and exploration of gases produced in the rumen, future work should also evaluate the use of NAP for adequately holding other gases, such as H_2_, N_2_, N_2_O, and H_2_S, to ensure they can be accurately characterized with this material type.

**Figure 2:**
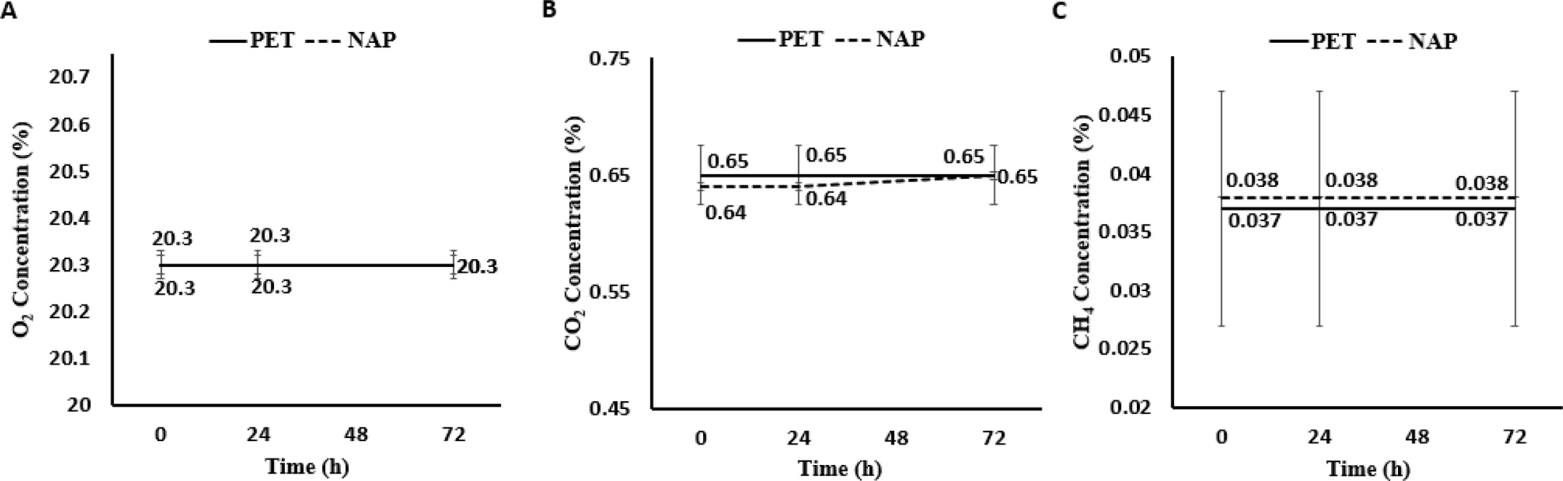
The percentage of O_2_ (A), CO_2_ (B), and CH_4_ (C) contained in a polyethylene terephthalate (PET) and multilayer fabrication (NAP) containing nylon, polyethylene, and aluminum-coated polyethylene plotted over time. No effect of bag material (*P* ≥ 0.36), time (*P* ≥ 0.69), or interaction between bag material and time was observed for any gas (*P* ≥ 0.61). Error bars indicate SEM.

Overall, results of the experiments indicate that relative to PET, PF gas bag material influences the composition of O_2_ and CO_2_ of stored respiratory gases within 24 h of sample collection. Thus, ensuring gas samples are analyzed within 24 h is crucial for the characterization of respiratory gases. No difference was observed over time for PET relative to a multilayered bag with aluminum coating. The current evaluation is limited in material types and gas storage lengths but provides material types which are suitable for characterization of respiratory gases included in the Brouwer (1965) equation given timely gas analysis. Thus, in the endeavor to characterize ruminant respiratory gases, future research should evaluate longer term storage of the samples and different volatile and nonvolatile breath components.
